# Correlation of computer-based test anxiety with medical students’ performance before, during and after assessments

**DOI:** 10.12669/pjms.38.3.4989

**Published:** 2022

**Authors:** Abida Shaheen, Fahad Azam, Muhammad Waqas Rabbani, Nosheen Kazmi

**Affiliations:** 1 Abida Shaheen, MBBS, M.Phil., Ph.D. Professor, Pharmacology & Therapeutics, Shifa College of Medicine, Shifa Tameer-e-Millat University, Islamabad, Pakistan; 2 Fahad Azam, MBBS, M.Phil., Ph.D. Associate Professor, Pharmacology and Therapeutics, Shifa College of Medicine, Shifa Tameer-e-Millat University, Islamabad, Pakistan; 3 Muhammad Waqas Rabbani, MBBS, M.Phil. Assistant Professor, Behavioral Sciences, Shifa College of Medicine, Shifa Tameer-e-Millat University, Islamabad, Pakistan; 4 Nosheen Kazmi, MBBS, MRCP. Speciality Registrar, Psychiatry, CAMHS, Pennine Care NHS Foundation Trust, Manchester, UK

**Keywords:** Anxiety, Students, Assessment, Formative

## Abstract

**Objectives:**

To explore the correlation of anxiety levels of medical students measured before, during and after assessments with their performance in formative assessments.

**Methods:**

A repeated measures cross-sectional study was conducted at Shifa College of Medicine, Islamabad with data collection at three points in time, from April-May 2020. A validated test anxiety questionnaire was used to assess anxiety. Eighty-two medical students recorded their responses on the questionnaire before, during and after three consecutive formative assessments in an integrated module. Relevant statistical tests were applied to investigate the correlation of anxiety levels at different stages with assessment scores.

**Results:**

The mean scores of anxiety measured before, during and after the three consecutive formative assessments were 29.78±7.77, 28.0±8.88 and 26.11±7.83, respectively. The difference of means of anxiety measured at different stages during assessments was statistically significant (p<0.001). The negative correlation between anxiety level and academic scores of female students was statistically significant (p=0.05).

**Conclusion:**

Fluctuation in anxiety scores at different stages of assessments affects academic performance. Identification of the effect of anxiety may help improve the academic performance of medical students.

## INTRODUCTION

There has been a worldwide paradigm shift from face-to-face to online computer-based teaching and assessment in the wake of the Covid-19 pandemic.^1^ The recommendations for Computer-Based Assessments (CBAs) in undergraduate medical education have been provided by the Association of Medical Education approximately two decades back.^2^,^3^ CBA is described as the use of information technology for online assessments requiring equipment such as computers, laptops and smartphones with a source of internet connectivity for designing, presenting and reporting student activities, scores, and feedbacks.^4^ Various platforms are available to conduct CBAs and each one has its own set of unique features and limitations.^5^ Recently, many features have been introduced to minimize the use of unfair means in assessments; however synchronous online proctoring has the potential to trigger anxiety in students.^6^,^7^

Test-related anxiety is a common phenomenon, ranging from 25-40% in undergraduate medical students worldwide.^8^ Various factors such as gender, extensive workload and late hour studies contribute to test anxiety; however, further research is required to explore the effects of anxiety on the students’ performance in synchronous computer-based assessments.^9^,^10^

The present study aimed to explore the anxiety levels of the students from the start to the end of the assessments and the relationship between these phases and exam performance.

## METHODS

A cross-sectional study was conducted with data collection at three points in time from April-May 2020. Third-year medical students of Shifa College of Medicine, Islamabad were recruited after taking approval from the Institutional Review Board (IRB#1004-279-2018). Informed consent from the participants was taken for each stage separately. Only those students were included who consented and participated in all three online assessments and recorded their responses on the anxiety questionnaire. A validated online test anxiety questionnaire by Nist and Diehl was used.^11^ This questionnaire has two parts; part A consists of demographic data; part B includes a five-point Likert scale (from “Never” to “Always”) with ten statements to explore how often participants experience the feeling described in each account.^11^ Each item received a weighting of 1-5, with 5 indicating high anxiety.^11^ The questionnaire score ranges from 10-50; a score >35 indicates unhealthy anxiety, a score of 20-35 denotes healthy anxiety and <20 shows low anxiety.^11^

The data were collected using “Google Forms” in three stages during MCQs-based formative assessments in the Neurosciences module. The anxiety questionnaire was embedded within MCQs to address maturation bias. Furthermore, to minimize the test sensitisation, the anxiety questionnaire was carefully timed for the first five minutes for the first stage, during the assessment for the second stage, and immediately after the assessment for the third stage for a total duration of 30 minutes for each assessment. MCQs in all three assessments were designed from the same module with an equal level of difficulty to ensure the methods’ internal validity.

Results of the assessments and participants’ responses to the anxiety questionnaire were compiled on the Excel sheet and data analysis was done on SPSS version 23. Repeated measure ANOVA and Pearson’s correlation were applied considering p-value ≤ 0.05 significant.

## RESULTS

A total of 104 students in three consecutive formative assessments responded to at least one of the anxiety tests taken during the three stages; 82 participants completed all three test anxiety questionnaires along with the formative assessments. The mean age of participants was 21.26±1.23 years. Shapiro-Wilk test was applied to average academic score and anxiety scores at all three stages before applying the inferential statistical tests and was found to be normally distributed. (Table-I) Repeated measure ANOVA (Bonferroni) was applied to measure the difference in anxiety scores at different stages. Multivariate analysis showed significance before, during and after the formative assessments (p<0.001). The pairwise comparison of the anxiety recorded before and during the assessments showed no statistical significance with a mean difference of 1.78 (p=0.21). However, the difference of anxiety means of 3.67 before and after the assessments was statistically significant (p=0.016); similarly, the mean difference between anxiety during and after the online assessment was 1.89 with high statistical significance (p=0.002). (Table-I).

**Table-I:** Descriptive data and anxiety levels of the participants during three stages of formative assessments.

	Assessment-1	Assessment-2	Assessment-3	p-value

	Pre-Assessment Anxiety	Anxiety during assessment	Post-Assessment Anxiety	
Males(%)	42.3	44	41	-
Females(%)	57.7	56	59	-
Number of Questions	35	37	32	-
Percentage score in assessment (Mean±SD)	85.27±16.90	84.22±9.40	80.49±9.62	-
Anxiety score (Mean±SD)	29.78±7.77	28.00±8.88	26.11±7.83	<0.001
Students with no anxiety (%)	10.8	16.2	24.3	-
Students with healthy anxiety(%)	66.2	63.5	62.2	-
Students with high anxiety(%)	23.0	20.3	13.5	-

**Fig.1:**
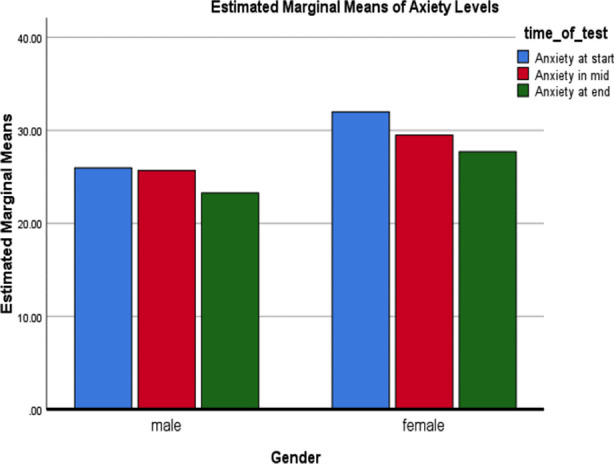
Effect of gender on anxiety score during different stages of assessments.

The anxiety scores in male students before, during and after formative assessments were 26.15±6.10, 25.63±10.40 and 23.56±7.27, respectively. Female students showed anxiety scores of 31.87±7.91, 29.36±7.67 and 27.57±7.83 before, during and after the formative assessments, respectively as shown in Fig.1. A comparison of anxiety scores before the formative assessment between male and female students demonstrated statistical significance (p=0.002); similarly, the difference in the anxiety scores of male and female students after the formative assessment was also significant (p=0.033). However, the comparison of anxiety scores between the two genders did not show statistical significance during the formative assessment (p=0.082).

The effect of gender on the level of anxiety before, during and after the assessment was measured by repeated measures ANOVA and demonstrated no statistical significance in males with a p-value of 0.095, however, anxiety scores in female students showed significance with a p-value <0.001 (Wilks’ Lambada) (Fig.1).

Pearson’s correlation and linear regression analysis were applied to measure anxiety with assessments performance. There was a negative correlation between anxiety level and academic scores; however, it was not statistically significant except in anxiety recorded during the assessment in females (p=0.05). (Table-II).

**Table-II:** Pearson’s correlation and linear regression analysis of anxiety scale with assessments’ performance.

Groups of Students	R	Β	Adjusted r^2^	p-value
Pre-assessment anxiety in males	0.30	0.775	0.056	0.12
Pre-assessment anxiety in females	-0.131	-0.295	-0.005	0.380
Pre-assessment anxiety in all students	-0.009	-0.019	-0.014	0.940
Anxiety during assessment in males	0.084	0.083	-0.033	0.677
Anxiety during assessment in females	-0.279	-0.327	0.57	0.05
Anxiety during assessment in all students	-0.10	-0.106	-0.004	0.398
Post-assessment anxiety in males	0.019	0.029	-0.040	0.924
Post-assessment anxiety in females	-0.076	-0.080	-0.016	0.611
Post-assessment anxiety in all students	0.039	0.049	-0.012	0.738

## DISCUSSION

Computer-based assessments (CBAs) have gained popularity to assess academic performance due to the recent advancements in online education and the current pedagogical shift towards online education during the COVID-19 pandemic. CBAs have been found to have a positive impact on learning and the academic performance of medical students.^12^,^13^ Limited data is available to explore anxiety levels during different stages of online assessments and their effect on academic performance in medical students.

Our results showed that students with high anxiety had lower scores but these results were not statistically significant. Our findings are consistent with previous studies showing an association of high trait anxiety with low scores in assessments. Studies conducted in Iran did not show a significant association between academic performance in assessments and test anxiety;^14^,^15^ however, other studies reported a significant association of test anxiety with reduced academic performance.^16^-^18^ These findings imply that various factors such as early education background, level of reward, motivation, and emotional quotient could influence academic achievement in addition to test anxiety.^19^

According to our results, more than 50% of students had a healthy level of anxiety in all formatives. A significant difference was found among the mean anxiety scores before, during and after formative assessment. Our findings are not in agreement with another study in which the majority of subjects had high levels of test anxiety.^20^ This difference could be attributed to several factors influencing anxiety such as different contents, format and conditions of the assessments.

Results of our study revealed a significant difference between levels of test anxiety and gender. Female students had a significantly higher level of test anxiety just before and after the test. However, test anxiety was not significantly related to gender and mean levels of anxiety during the test. Our findings are consistent with another study demonstrating gender to be a significant factor in test anxiety among students; though anxiety levels at different stages of assessments have not been explored yet.^21^ Previous studies have also reported a higher prevalence of test anxiety in female students.^22^,^23^

These findings can be explained with Huberty’s statement that although everyone worries occasionally, excessive worry may affect academic functioning and contribute to feelings of loss of control and depression, especially in female students.^24^ On the contrary, our findings contradict a study conducted in India reporting higher test anxiety in males.^25^ This discrepancy might be due to variation in the study region and participants since the study sample had included high school students with a higher number of male participants.

A gender-wise comparison revealed that male students with high anxiety levels performed better but this result was not statistically significant. However, females in the second formative showed a significant correlation between anxiety and test scores. The possible reason for this strong correlation could be the tendency of females to study excessively before exams creating fatigue and over-exertion which may negatively affect performance in assessments.^8^

Test anxiety is a serious psychological problem and can severely affect the academic performance of many students. Lack of the evaluation of the mental health of the participants is a major limitation of the present study. Gender role in test anxiety ought to be investigated in relation to all confounding variables. Similar studies should be conducted on students studying in different years of medical school and in other provinces of the country to make necessary interventions and comprehensive plans regarding online exams, enabling students to perform to the best of their mental capability.

## CONCLUSION

In summary, there is a significant difference between anxiety levels measured at different stages of assessments. A significant correlation exists between anxiety levels and the academic performance of female medical students. Students’ anxiety in online exams can be decreased by using new educational strategies such as familiarization with electronic technology in learning and assessments. Future studies are needed to evaluate and compare the effect of different types of technologies such as tablets or mobile phones on test anxiety and performance. The results of this study could be helpful for academic advisers and planners, developers of education systems, and mental health planners.

### Authors Contribution:

**AS:** Conceived & designed study, collected data, conducted statistical analysis, drafted manuscript.

**FA:** Conceived & designed study, collected data, conducted statistical analysis, drafted manuscript.

**MWR:** Conducted statistical analysis, reviewed and approved the final manuscript.

**NK:** Conceived study, reviewed and approved the final manuscript.

All authors are responsible and accountable for the accuracy and integrity of the work.

## References

[1] Khan RA, Jawaid M (2020). Technology Enhanced Assessment (TEA) in COVID 19 Pandemic. Pak J Med Sci.

[2] Al-Amri S, Ali Z (2016). Systematic Review of Computer Based Assessments in Medical Education. Saudi J Med Med Sci.

[3] Dennick R, Wilkinson S, Purcell N (2009). Online eAssessment:AMEE guide no. 39. Med Teach.

[4] Kolagari S, Modanloo M, Rahmati R, Sabzi Z, Ataee AJ (2018). The Effect of Computer-based Tests on Nursing Students'Test Anxiety:a Quasi-experimental Study. Acta Inform Med.

[5] Luo L, Cheng X, Wang S, Zhang J, Zhu W, Yang J (2017). Blended learning with Moodle in medical statistics:An assessment of knowledge, attitudes and practices relating to e-learning. BMC Med Educ.

[6] Lilley M, Meere J, Barker T (2016). Remote Live Invigilation:A Pilot Study. J Interact Media Educ.

[7] Kolski T, Weible J (2018). Examining the relationship between student test anxiety and webcam based exam proctoring. Online J Distance Learn Adm.

[8] Tsegay L, Shumet S, Damene W, Gebreegziabhier G, Ayano G (2019). Prevalence and determinants of test anxiety among medical students in Addis Ababa Ethiopia. BMC Med Educ.

[9] Khoshhal KI, Khairy GA, Guraya SY, Guraya SS (2017). Exam anxiety in the undergraduate medical students of Taibah University. Med Teach.

[10] Woldeab D, Brothen T (2019). 21st Century Assessment:Online Proctoring, Test Anxiety, and Student Performance. Int J E-Learning Distance Educ.

[11] Nist P, Diehl M (1990). PHCC Test Anxiety Questionnaire.

[12] Azam F, Irshad K, Shaheen A, Moin H, Javed N, Ahmer H (2021). Online versus Conventional Paper Based Formative Assessment:Do They Predict Summative Scores?. Pak J Physiol.

[13] Azam F, Shaheen A, Irshad K, Javed N, Ata M (2018). Trends of undergoing formative assessment in undergraduate medical students. J Shifa Tameer Millat Univ.

[14] Kashfi SM, Jeihooni AK, Kashfi SH, Yazdankhah M (2014). The relationship between test anxiety and educational performance among the students at school of health and nutrition, Shiraz university of medical sciences in 2011. J Contemp Med Edu.

[15] Cheraghian B, Fereidooni-Moghadam M, Baraz-Pardejani S, Bavarsad N (2008). Test anxiety and its relationship with academic performance among nursing students. Knowl Heal.

[16] Gunawardena S, de Zoysa P, Jayasinghe S, Manathunge A, Alles H, Shenoy V (2017). Selected correlates associated with test anxiety among 14-16 year olds in a Colombo district school. Sri Lanka J child Heal.

[17] Sideeg A (2015). Test anxiety, self-esteem, gender difference, and academic achievement:the case of the students of medical sciences at Sudanese universities:a mixed methods approach. Br J Arts Soc Sci.

[18] Balogun AG, Balogun SK, Onyencho CV (2017). Test anxiety and academic performance among undergraduates:the moderating role of achievement motivation. Span J Psychol.

[19] Steinmayr R, Crede J, McElvany N, Wirthwein L (2016). Subjective Well-Being, Test Anxiety, Academic Achievement:Testing for Reciprocal Effects. Front Psychol.

[20] Clark JW, Fox PA, Schneider HG (1998). Feedback, test anxiety and performance in a college course. Psychol Rep.

[21] Nunez-Pena MI, Suarez-Pellicioni M, Bono R (2016). Gender Differences in Test Anxiety and Their Impact on Higher Education Students'Academic Achievement. Procedia –SocBehav Sci.

[22] Farooqi YN, Ghani R, Spielberger CD (2012). Gender differences in test anxiety and academic performance of medical students. Int J Psychol Behav Sci.

[23] Chapell MS, Blanding ZB, Silverstein ME, Takahashi M, Newman B, Gubi A (2005). Test anxiety and academic performance in undergraduate and graduate students. J Educ Psychol.

[24] Huberty TJ (2009). Test and performance anxiety. Princ Leadersh.

[25] Salend SJ (2012). Teaching students not to sweat the test. Phi Delta Kappan.

